# Artificial intelligence in systemic diagnostics: Applications in psychiatry, cardiology, dermatology and oral pathology

**DOI:** 10.6026/973206300210105

**Published:** 2025-02-28

**Authors:** Jaden Penhaskashi, Jonah Danesh, Arya Naeim, Josh Golshirazi, Justin Hedvat, Francesco Chiappelli

**Affiliations:** 1 Division of West Valley Dental Implant Center, Encino, California 91316; 2 University of California, Los Angeles; 3 Independent Researcher, UCLA Health; 4 Center for the Health Sciences, UCLA, Los Angeles, California and Dental Group of Sherman Oaks, California 91403

**Keywords:** Artificial intelligence, machine learning, deep learning, convolutional neural networks, neural networks, single nucleotide polymorphisms, genetic biomarkers, systemic inflammation, diagnostic imaging, functional MRI, cardiac magnetic resonance, fractional amplitude of low-frequency fluctuations, cardiovascular disease, digital biomarkers, psychiatry, cardiology, dermatology, oral pathology

## Abstract

The integration of Artificial Intelligence (AI) in to the field of medicine is offering a new-age of updated diagnostics, prediction and treatment across multiple fields, addressing systemic disease including viral infections and cancer. The fields of Oral Pathology, Dermatology, Psychiatry and Cardiology are shifting towards integrating these algorithms to improve health outcomes. AI trained on biomarkers (*e.g.* salivary cf DNA) has shown to uncover the genetic linkage to disease and symptom susceptibility. AI-enhanced imaging has increased sensitivity in cancer and lesion detection, as well as detecting functional abnormalities not clinically identified. The integration of AI across fields enables a systemic approach to understanding chronic inflammation, a central driver in conditions like cardiovascular disease, diabetes and neuropsychiatric disorders. We propose that through the use of imaging data with biomarkers like cytokines and genetic variants, AI models can better trace the effects of inflammation on immune and metabolic disruptions. This can be applied to the pandemic response, where AI can model the cascading effects of systemic dysfunctions, refine predictions of severe outcomes and guide targeted interventions to mitigate the multi-systemic impacts of pathogenic diseases.

## Background:

Disease, whether viral or cancerous, impacts immune, inflammatory and metabolic systems - in turn disturbing the individual's homeostasis. AI has recently emerged as a new front for addressing these challenges, leveraging modeling algorithms on both existing and novel datasets to advance diagnostics, prediction and treatment. During the COVID-19 pandemic, enhanced epidemiological surveillance and diagnostic support were made possible through such 'intelligent' algorithms. AI-analyses on airline ticketing and social media data allowed for improved contact tracing efficiency and Machine Learning was used to interpret imaging data to detect COVID-19-related lung damage [1, 2]. Beyond the respiratory system, COVID-19 affects the physiology of the heart, skin, as well as oral cavity, highlighting the need for systemic approaches to understanding disease impacts [3, 4-5]. Similarly, cancer affects multiple systems through inflammation and cachexia, which impairs skeletal, digestive and cardiac functions [6]. The aim of this paper is to explore the potential of AI in addressing such systemic challenges in health, focusing on applications in Oral Pathology, Cardiology, Dermatology and Psychiatry.

## Oral pathology:

## Detection and prognosis of oral cancer:

Convolutional Neural Networks (CNNs) have begun to diagnose squamous tongue carcinoma as well as estimate metastasis risk by analyzing changes in texture, shape and size in tumor Computed Tomography scans [7]. Additionally, histopathological analysis of tissue biopsies, including the site's neutrophil-to-lymphocyte ratio, enables these models to distinguish between benign and malignant oral tissue [8]. Saliva biopsies separately contain genetic and epigenetic markers closely linked to cancer progression-datasets already being integrated into intelligent models for increased diagnostic and risk prediction [9-10]. Complementing these approaches, certain deep learning models analyze factors such as Human Papillomavirus (HPV) status, tumor stage and lymph node involvement to tailor plans for patients with head and neck cancer. Such models predict how effective radiation therapy would be for the individual and whether additional chemotherapy is necessary [11]. This is especially important for older patients, where chemotherapy outcomes are less predictable and the avoidance of unnecessary treatments is crucial.

## Advancements in dental imaging:

Object recognition Convolutional Neural Networks tailored for analyzing periapical X-ray images have significantly improved the detection accuracy of carious lesions and other pathologies, such as bone loss from periodontal disease and root fractures [12]. Similar to periapical X-rays, Cone Beam CT provides high-resolution 3D imaging of bone structure, volume and tissue depth, essential for implant planning. Convolutional Neural Networks automatically identify and align anatomical markers, significantly reducing human error, optimizing implant stability catered to the patient's anatomy [13, 14].

## Disease progression analysis through biomarkers:

Biomarkers and immunological data, such as leukocyte counts and interleukins have been incorporated in Convolutional Neural Networks and shown to differentiate between aggressive and chronic periodontitis with high accuracy [15]. Cytokines and enzymes detected in saliva and gingival fluid help the algorithms to identify periodontal inflammation and its correlation with cardiovascular health and diabetes [16]. Oral bacteria such as Porphyromonas gingivalis are closely linked to systemic inflammation and complications like cardiovascular disease [17]. Integrating bacterial data with inflammatory biomarkers represents a critical next step for AI in differentiating periodontal diseases from systemic inflammatory conditions and predicting predisposition to viral infections. Similarly, data from greasy tongue coatings are being used to train CNN models. Through analyzing coating thickness, color and pattern, similar health risks are accurately classified, including heart disease, gastroenteropathy and viral infections [18].

## Dermatology:

## Diagnosis and prognosis of skin lesions:

Skin lesion detection and categorization is making rapid progress through the application of intelligent algorithms. Convolutional Neural Networks identify minute lesion characteristics including color, texture and shape, outperforming traditional techniques. Diagnostic accuracy comparable to that of board-certified dermatologists for conditions like melanoma and basal cell carcinoma has been achieved by models trained on clinical photographs, allowing for distinction between benign and malignant lesions [19]. Overall, this can lead to higher accessibility of reliable diagnostic tools in underserved areas, reducing reliance on specialists and improving care delivery. As clinical datasets grow in quantity and specificity, these advancements are expected to extend to rare skin conditions and further enhance diagnostic precision [20].

## Predictive models for skin cancer susceptibility:

Machine Learning (ML) models trained on genetic and lifestyle factors identify heightened susceptibility to melanoma and other skin cancers. Customized monitoring plans for patients with specific genetic markers or environmental exposures facilitate early detection and reduce the need for aggressive treatments after disease onset [21].

## Management of chronic skin conditions:

Incorporating datasets, including DNA sequencing, biomarkers and patient histories, enhances the accuracy of dermatological diagnosis. This reduces reliance on traditional trial-and-error methods, leading to fewer side effects and enabling effective, targeted therapies [22, 23]. Machine Learning has improved the management of chronic skin illnesses, with models predicting disease progression in conditions such as psoriasis and eczema [20]. Forecasting flare-ups based on patient data allows the clinician to adjust the treatment-plan in advance, rather than solely acting on symptom severity.

## Psychiatry:

## Genetic variation in psychiatric conditions:

Data analysis of genome-wide association studies through AI techniques have discovered Single Nucleotide Polymorphisms (SNPs) and risk alleles which correlate to specific neurological diseases within patient cohorts of similar ethnic ancestry. A recent CNN has successfully identified schizophrenia patients through analysis of Single Nucleotide Polymorphisms in the HTR2A and DRD3 genes- known to be linked to dopamine and serotonin receptors [24]. Beyond SNPs, rare structural variations in genes have recently been associated with bipolar disorder by a machine learning algorithm [25]. There is a clear importance of diverse genomic data sources, in the pursuit of AI-driven diagnostic accuracy, especially within the field of mental health.

## Psychiatric diagnosis through imaging:

CNN algorithms have also been paired with EEG and neuroimaging datasets for the improved diagnosis of sleep stages, major depression and schizophrenia, with up to 90% accuracy [26-27]. Data derived from functional MRIs can be especially useful, including fractional Amplitude of Low- Frequency Fluctuations (fALFF), a measure of the resting-state brain activity. Recently applied Support Vector Machines (SVMs) have discovered a correlation between increased fractional Amplitude of Low- Frequency Fluctuations across brain regions during an individual's first episode of major depression [28]. Fractional Amplitude of Low- Frequency Fluctuations data can also be integrated with metrics such as regional homogeneity with ensemble models which can further progress diagnostic precision for schizophrenia [29].

## Personalized treatment strategies:

AI-driven models have also identified promising digital biomarkers for advancing mental health diagnostics with certain populations. Location data, call activity and screen usage has assisted the ML-diagnosis of depression in adolescents, while voice is a candidate marker for predicting depression in individuals with Parkinson's disease [30, 31].

Treatment for psychiatric conditions traditionally includes medication, speech therapy and cognitive behavioral therapy. The public availability of Large Language Models (LLMs) improves accessibility and adaptability of these therapies. Case in point, recent voice-based coaches have been modeled as problem-solving therapists for individuals with depression, with evidence of enhancing problem-solving skills and reducing avoidance behavior [32].

## Cardiology:

## Echocardiography and cardiac imaging:

Echocardiography, a cost effective and non-invasive tool for assessing blood flow and identifying abnormalities, has traditionally had its accuracy limited by operator variability [33]. Through interpreting critical benchmarks for assessing heart function such as ejection fraction, endocardial excursions and myocardial thickening, Machine Learning models have improved such evaluation consistency in heart failure and ischemic coronary artery disease diagnoses [33, 34-35]. In parallel, Cardiac Magnetic Resonance (CMR), another non-invasive imaging tool, has faced challenges such as long acquisition times, which is particularly challenging for patients who struggle with breath-holding during scans. Through deep learning assisted improved accuracy in measuring left ventricular parameters and wall thickness, faster image reconstruction is enabled without sacrificing detail [33, 34, 35-36].

## Genetic indicators of cardiovascular risk:

ML algorithms have also identified transcriptomic biomarkers linked to Cardiovascular Disease (CVD), including RN7SL593P, associated with platelet function, reflecting the well-established links between the cardiovascular and immune systems [37]. Inflammation, driven by cytokines and immune cells, is central to Cardiovascular Disease development, particularly in atherosclerosis and plaque vulnerability. This understanding has led to anti-inflammatory treatments, some of which reduce the risks of myocardial infarction and stroke [38]. Further exploration of these genetic associations could deepen understanding of the inflammatory mechanisms which underlie cardiac disease.

## Conclusion:

AI has demonstrated its ability to address challenges within physiological systems, such as immune, inflammatory and metabolic disruptions, which are interconnected in feedback loops that amplify dysfunction. For instance, chronic inflammation-a protective immune response that transitions into systemic dysfunction-is associated with conditions like cancer, fatty liver disease and cardiovascular disease [39]. System-specific effects include sustained inflammatory signaling exacerbating metabolic strain in psoriasis, impaired glucose regulation in diabetes and cytokine-driven inflammation contributing to Cardiovascular Disease and neuropsychiatric conditions like schizophrenia and major depressive disorder [16, 20, 37, 40 and 41]. AI's current applications target specific systems within individual fields of health, with advances driven by imaging, genetic datasets and systemic biomarkers. Genetic datasets are particularly helpful in uncovering molecular drivers that predispose individuals to systemic dysfunctions. From SNP associations of patients with neuropsychiatric disorders [24] to the linked DNA methylation patterns of liquid biopsies to cancer [10]. Radiological tools help visualize the functional effects of these negative effects, as seen in persistent skin lesions such as eczema, serving as visible indicators of chronic immune and metabolic imbalances, often linked to systemic fatigue [20]. Advances in AI-enhanced cardiac imaging improve resolution and consistency, deepening understanding of cardiovascular disease progression and its links to chronic inflammation [37, 38- 39]. The integration of imaging and genetic datasets across fields can provide a comprehensive and novel view of disease. Imaging tools detect structural changes from immune and inflammatory activity, while genetic algorithms in turn contextualize these findings based on population susceptibility. Biomarkers can further refine diagnostics and treatments by identifying imbalances in homeostasis, such as speech patterns linked to mental health disorders which cause such immune disruptions [31, 32, 40 and 41].

## The future applications of AI in pandemic response:

Pandemics highlight the critical need for this synthesis of the datasets to better understand and mitigate the multi-systemic impacts of pathogenic diseases. During viral outbreaks, dermatological manifestations such as inflammatory skin lesions can serve as early indicators of immune over activation, which AI-enhanced imaging tools can detect and monitor to predict broader systemic complications [20]. Similarly, cytokine biomarkers in oral fluids and links to cardiovascular inflammation offer non-invasive insights into multi-organ vulnerabilities heightened by viral infections [15, 16]. The AI-driven analysis of echocardiographic and MRI data can identify myocardial inflammation or early cardiac dysfunction, common precursors to severe outcomes in systemic viral infections [38]. Genetic tools further enable stratification of patient populations by identifying susceptibility markers such as Single Nucleotide Polymorphisms linked to immune and metabolic dys-regulation, refining the prediction of severe outcomes and enabling targeted interventions [24]. Through integrating these inputs, AI can model the cascading effects of systemic inflammation and metabolic dysfunction, offering a holistic view of disease progression.
